# Mitochondrial pharmacology turns its sights on the Ca^2+^ uniporter

**DOI:** 10.1038/cddiscovery.2017.64

**Published:** 2017-09-25

**Authors:** Nina M Storey, David G Lambert

**Affiliations:** 1 Department of Molecular and Cell Biology, University of Leicester, Leicester LE1 9HN, UK; 2 Department of Cardiovascular Sciences, University of Leicester, Division of Anaesthesia, Critical Care and Pain Management, Leicester Royal Infirmary, Leicester LE2 7LX, UK

To use a well-known phrase, mitochondria are the ‘power house of the cell’ and produce ATP, the energetic currency of life.^1^ Although this is a vital role, mitochondria also have many other crucial roles including cell death pathways such as collapse of the mitochondrial membrane potential by opening the mitochondrial permeability transition pore (mPTP). These roles are profoundly affected by the entry of calcium into the mitochondria. Mitochondrial calcium increases the likelihood of mPTP opening, regulation of metabolic rate by activation of metabolic enzymes (dehydrogenases), reactive oxygen species production, signalling (Ca-calmodulin) and influencing the pattern of global calcium oscillations in response to receptor stimulation.^2^

Calcium uptake into mitochondria was first discovered in the 1960s by measuring radiolabelled calcium influx into isolated mitochondria from rat kidney homogenates.^3,4^ The biochemical research that followed took advantage of ruthenium red and lanthanum compounds which are potent inhibitors of calcium flux through calcium selective pores. The problem with these compounds is that they are not cell permeable and therefore useful only in studying isolated mitochondria or mitoplasts (mitochondria without outer membranes). This has limited the interpretation of the results to the level of the mitochondria rather than the context of the cell or the whole organ or indeed the organism.

Exploration of the more cellular aspects of mitochondrial calcium uptake has been possible through use of aequorin, a calcium-sensitive luminescent protein from jellyfish that detects changes in calcium concentrations in bioluminescence assays. Mitochondrially tagged aequorin has been exploited to report changes in intramitochondrial calcium concentrations and over the years has been of enormous value in understanding mitochondrial calcium uptake. In terms of overall calcium concentration, it is worth noting that mitochondria accumulate orders of magnitude higher levels than free cytosolic calcium concentrations and the driving force for this staggering calcium accumulation is the mitochondrial membrane potential (−180 mV) generated by the respiratory chain proton gradient.^5^ It has emerged that the mitochondrial calcium uptake mechanism appeared to have a relatively low affinity for calcium which supports the idea that microdomains must reach much higher local calcium concentrations for calcium uptake to occur. What has also proved interesting is that at rest mitochondria do not accumulate calcium and that uptake occurs after physiological receptor stimulation, such as leukotriene receptor activation in immune cells.^6^

Almost 50 years after discovering that mitochondria accumulate calcium, a significant change in our understanding occurred from an ingenious combination of both proteomics and genomics which revealed the identity of a mitochondrial calcium uniporter (MCU). This uniporter was found to be responsible for the radiolabelled calcium uptake observed half a century earlier.^7,8^ Finding the uniporter was achieved using the Mitocarta database. The database is a compilation of mouse genes that were found by mass spectroscopy analysis of isolated mitochondria and from other proteins known to have a mitochondrial target sequence. This database was mined for candidate ruthenium red sensitive mitochondrial calcium uptake proteins which appear in the genome phylogenetically after yeast (which do not have a MCU).

This was an exciting time as it marked a shift from the biochemical approaches into molecular approaches such as silencing RNA, overexpression, dominant negatives and knockout mice. There has indeed been an explosion of deeper understanding of MCU, including the emergence of many auxiliary regulatory proteins to form what is now known as the ‘mitochondrial uniporter complex’. This is perhaps no surprise to those studying mitochondrial calcium uptake as the first protein identified in the search was an integral member of the MCU complex, mitochondrial calcium uptake protein 1. It has calcium sensitive domain to detect calcium concentration changes, making it the gatekeeper of mitochondrial calcium uptake but without any other characteristics of a uniporter or channel it was clearly not the protein responsible for calcium influx (Figure 1).

A number of drugs targeted to the mitochondria have been developed such as Mito Q^9^ an antioxidant acting to ‘mop up’ reactive oxygen species and prevent damage. This may prove to be highly beneficial in cardiovascular diseases.^10^ The paper described here may mark the dawn of the next era of research into mitochondrial calcium because it describes a novel, cell permeable and specific mitochondrial calcium uptake inhibitor.^11^ Perhaps this will provide the next step change in our understanding of the function and role of MCU and its complex. Kon *et al.*^11^ have used high-throughput screening to identify a cell permeable drug DS16570511, which inhibits mitochondrial calcium entry not only in isolated mitochondria but also in cells and in the whole heart. Now the rapid effects of a cell permeable, fast-acting mitochondrial calcium uptake inhibitor can be observed in cells and tissues with the potential to provide invaluable insight into the roles and functioning of the MCU complex.

Kon *et al.*^11^ have screened a library to pull out compounds that inhibit mitochondrial targeted aequorin luminescence but did not inhibit a cytosolic aequorin luminescence. This compound inhibited mitochondrial calcium uptake while having no effect on mitochondrial membrane potential tested by the indicator JC-10. This is good evidence that DS16570511 is more likely to block calcium entry rather than being a compound that collapses the driving force for calcium entry. The advantage of using cell permeable drugs is that the fast-acting effects avoid the compensatory mechanisms that can often cloud interpretation of knockout studies. Most exciting of all is the ability to examine the effects of the rapidly acting drugs in cells and indeed in *ex vivo* organs and potentially *in vivo*. In the *ex vivo* Langendorff model, DS16570511 prevented calcium overload, which is usually observed when hearts are perfused with a high calcium concentration solution.

One caveat is that newly discovered drugs rarely remain truly specific for long as other effects at different sites gradually become apparent. Ruthenium derivatives such as ruthenium red and Ru360 have been potent and powerful tools but cell permeable small molecules are a much better proposition for research and potential therapeutics. Unfortunately, DS16570511 has a relatively low potency compared with ruthenium red but hopefully the emergence of small molecule drugs with a potency that matches the ruthenium compounds is just around the corner. It is also unclear whether DS16570511 binds to MCU or one of the multiple auxiliary subunits within the complex (Figure 1). In addition to not knowing the binding site of DS16570511, there is also no current indication of the molecular mechanisms of its action.

Despite the unknowns there are opportunities here to learn more and it will be fascinating to see how this drug will affect mPTP opening and cell death in heart ischaemia reperfusion injury models. Depending where the inhibitor binds, it may also reveal the nature of cross-talk between MCU complex members and molecular mechanisms of calcium uptake regulation. Perhaps, we can even look forward to understanding the nature of how the mircodomains are organised and cross-talk with mitochondrial associated membranes and other organelles such as the endoplasmic reticulum.

## Additional information

**Publisher’s note:** Springer Nature remains neutral with regard to jurisdictional claims in published maps and institutional affiliations.

## Figures and Tables

**Figure 1:**
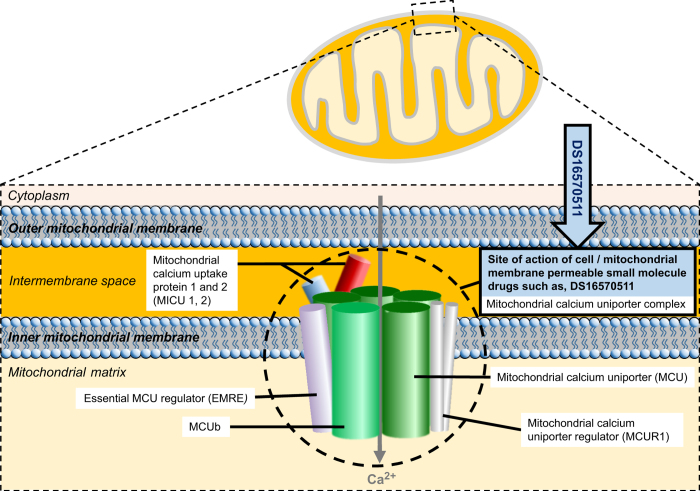
The site of action of small molecule drug DS16570511. The cell and mitochondrial membrane permeable small molecule drug DS16570511 acts at the mitochondrial calcium uniporter complex. This is a schematic representation of the mitochondrial calcium uniporter complex because the cross talk and interaction sites for all the regulatory components of the complex are not fully understood. MICU1 was the first member of the complex to be found, it has calcium sensitive domain and is a regulatory subunit. Next the pore forming subunit, the mitochondrial calcium uniporter was found. Other members of the MCU complex are reviewed elsewhere.^2^
